# Cervical Open-Door Laminoplasty for Myelopathy Caused by Ossification of the Posterior Longitudinal Ligament: Correlation Between Spinal Canal Expansion and Clinical Outcomes

**DOI:** 10.3390/jcm13226904

**Published:** 2024-11-16

**Authors:** Young-Il Ko, Young-Hoon Kim, Jorge Barraza, Myung-Sup Ko, Chungwon Bang, Byung Jun Hwang, Sang-Il Kim, Hyung-Youl Park

**Affiliations:** 1 Department of Orthopedic Surgery, Seoul St. Mary’s Hospital, College of Medicine, The Catholic University of Korea, Seoul 06591, Republic of Korea; 2 Department of Orthopedic Surgery, ABC Medical Center, Mexico City 01120, Mexico; jbarraza933@gmail.com; 3 Department of Orthopedic Surgery, Eunpyeong St. Mary’s Hospital, College of Medicine, The Catholic University of Korea, Seoul 03312, Republic of Korea

**Keywords:** cervical vertebrae, ossification of the posterior longitudinal ligament, spinal canal, myelopathy, laminoplasty

## Abstract

**Background/Objectives**: This study investigated the relationship between spinal canal expansion and clinical outcomes in patients with myelopathy due to ossification of the posterior longitudinal ligament (OPLL) who underwent cervical open-door laminoplasty. **Methods**: A retrospective study was conducted on 36 OPLL patients who underwent open-door laminoplasty between 2009 and 2021. Preoperative and two-year postoperative radiologic parameters, including bony canal area (BCA) and spinal canal area (SCA), were measured. Clinical outcomes were assessed using the Numerical Rating Scale (NRS) for neck pain and radicular pain, the Neck Disability Index (NDI), and Japanese Orthopaedic Association (JOA) scores. **Results**: The mean expansion of BCA was 112.1 mm^2^ (47%) and SCA was 100.5 mm^2^ (64%). All clinical outcomes improved after surgery, although not statistically significant. JOA scores improved significantly in the severe group, while NDI and NRS-neck scores improved in the mild to moderate group. Significant correlations were found between improvements in NRS-neck and expansions of BCA (r = 0.533, *p* = 0.001) and SCA (r = 0.537, *p* = 0.001). NDI improvement was also associated with BCA expansion. No significant correlations were found between canal expansion and NRS-R, NRS-L, or JOA scores. **Conclusions**: Cervical open-door laminoplasty effectively increased the bony and spinal canal areas in patients with OPLL and myelopathy. In addition to improving myelopathy symptoms, this procedure may also improve neck pain and disability. Further research is needed to assess the long-term outcomes and to better understand these clinical improvements.

## 1. Introduction

Ossification of the posterior longitudinal ligament (OPLL) is a condition characterized by the replacement of the posterior longitudinal ligament (PLL) with heterotopic bone [1]. The prevalence of OPLL is 0.60% in South Korea and 3% in other Asian countries, with male-to-female ratios of 1.45:1 and 2:1, respectively [2,3]. Pathophysiologically, fibroblasts within the PLL are replaced by cartilaginous tissue, followed by endochondral ossification and replacement of the PLL with lamellar bone [1]. OPLL may be asymptomatic and is often found incidentally. When symptomatic, neck pain and radiating pain typically present first, with myelopathy symptoms usually progressing slowly [4]. Diagnosis is generally made through lateral radiographs showing an ossified mass posterior to the vertebral bodies, with further imaging with computed tomography (CT) and magnetic resonance imaging (MRI) recommended for preoperative planning [5].

Surgical treatment is the only definitive method to decompress the spinal canal and can be achieved via anterior or posterior approaches to the cervical spine. Anterior decompression often involves corpectomy followed by direct removal of the pathology or the floating technique, while the posterior approach typically involves indirect decompression through laminoplasty or laminectomy with fusion [6,7]. Open-door laminoplasty, an indirect decompression technique, expands the dimensions and area of the spinal canal by freeing the laminae on one side and securing them in a more posterior position. Numerous studies have reported sufficient decompression and neurological recovery following laminoplasty [8,9].

Additionally, some reports suggest the radiological effectiveness of surgical decompression and subsequent neurological recovery, as measured by the spinal canal dimensions and the percentage of occupancy after the procedure [10,11]. However, few studies have analyzed the relationships between increased spinal canal area and various clinical symptoms, including not only neurological recovery but also axial or radicular pain.

This study aimed to measure and compare the bony canal area, spinal canal area, and OPLL before and after multilevel open-door laminoplasty in patients with cervical OPLL and myelopathy and to determine the relationships between these radiologic measurements and clinical outcomes.

## 2. Materials and Methods

This retrospective study included 36 patients who underwent surgery for cervical myelopathy due to OPLL between 2009 and 2021. The inclusion criteria were as follows: myelopathy associated with multilevel OPLL (involving two or more cervical levels), absence of cervical kyphosis, absence of dynamic instability, and a minimum follow-up period of more than two years post-surgery. Myelopathy symptoms included hand numbness, loss of fine motor function, bilateral paresthesia, impaired gait, lower extremity weakness, and urinary or fecal urgency and incontinence [12]. Exclusion criteria included myelopathy caused by cervical disc herniation or spondylosis, cervical kyphosis, previous cervical surgery, spine trauma, tumor, infection, or incomplete medical and radiographic records. All patients underwent a single surgical procedure and received conservative postoperative treatment, including pain management medication. This study was ethically approved by the institutional review board (KC21RISI0093).

### 2.1. Surgical Procedure

After general anesthesia and prone positioning, a posterior cervical approach was performed. The paravertebral muscles were detached from the spinous processes on both sides of the affected level. Once the spinous process and laminae were exposed, a high-speed burr was used to cut through one side of the lamina on the opening side and partially through the other side, preserving one cortex on the hinge side as part of the open-door laminoplasty technique [9]. A Medtronic mini-plate (Centerpiece, Medtronic, Minneapolis, MN, USA) was then applied and secured with one screw on the medial lamina and two screws on the lateral mass. Plates ranging in length from 10 mm to 12 mm were used. Previous studies have demonstrated that C3 laminectomy, when combined with multilevel open-door laminoplasty for OPLL, can effectively reduce postoperative axial neck pain [13,14]. C3 laminectomy offers distinct advantages over C3 laminoplasty: prevention of C2–3 bone fusion and preservation of posterior cervical musculature attachments [15]. Based on this evidence, our institution has implemented C3 laminectomy in conjunction with multilevel open-door laminoplasty as a standard surgical protocol since 2018. Intraoperative neuromonitoring with transcranial motor-evoked and somatosensory-evoked potentials was utilized during the surgery.

### 2.2. Radiologic Parameters

Cervical spine anteroposterior and lateral views, along with CT scans obtained before and after surgery, were analyzed. Radiographic measurements of cervical lordosis (CL) and cervical sagittal vertical axis (CSVA) were performed on neutral lateral views. CL was measured using the inferior endplate of C2 as the superior reference and the inferior endplate of C7 as the inferior reference. CSVA was determined by measuring the distance between a plumb line dropped from the center of the C2 body and the posterior superior corner of C7 [16].

**Figure 1:**
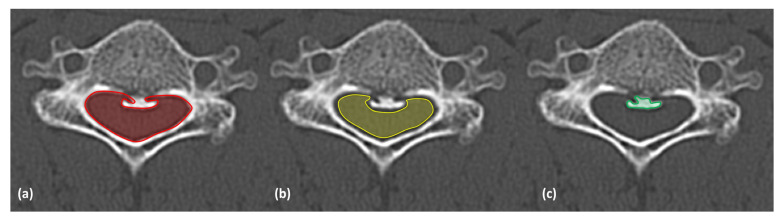
(**a**) Bony Canal Area (BCA) in red, defined by the posterior edge of the vertebral body, the pedicles, and the union of the laminae. (**b**) Spinal Canal Area (SCA) in yellow, bounded anteriorly by the posterior limit of the posterior longitudinal ligament (PLL) or ossification of the posterior longitudinal ligament (OPLL) and posteriorly by the inner edge of the ligamentum flavum. (**c**) OPLL in green, showing the outer boundary of the ossified posterior longitudinal ligament.

Radiographic measurements of cross-sectional areas of the bony canal and the spinal canal were performed by two independent observers, similar to the methodology used by Dong et al. [10]. The bony canal area (BCA) was delineated by the posterior border of the vertebral body, pedicles, and the union of both laminae. After surgery, the plate replaced the posterior border of the operated laminae. If one or both pedicles were not visible on the CT scan, the posterior margin of the nerve root was used as the border. The entire external border of the OPLL was delineated, and the area was measured. The spinal canal area (SCA) was defined anteriorly by the posterior margin of the PLL or OPLL and posteriorly by the inner margin of the ligamentum flavum (Figure 1). BCA, SCA, and the cross-sectional area of the OPLL were measured at all levels before surgery and two years after surgery.

### 2.3. Clinical Parameters

Clinical outcomes were evaluated using the Numerical Rating Scale (NRS) for neck pain (NRS-neck) and radicular pain in the right and left arms (NRS-right arm [NRS-R] and NRS-left arm [NRS-L]), the Neck Disability Index (NDI), and the Japanese Orthopaedic Association (JOA) score. These parameters were assessed preoperatively and two years postoperatively [17,18]. Patients were stratified according to their JOA scores; those with scores ≤ 11 were categorized as severe myelopathy, whereas those with scores ≥ 12 were classified as mild to moderate myelopathy [19]. Because postoperative neck pain has been reported as the most significant difference between C3 laminectomy and laminoplasty, the NRS-neck scores were compared between the C3 laminectomy group and the laminoplasty group through subgroup analysis [15].

### 2.4. Statistical Analysis

Statistical analyses were conducted using SPSS software version 24.0.0 (SPSS Inc., Chicago, IL, USA). A *p*-value of less than 0.05 was considered statistically significant. For the comparison of paired data, either the paired *t*-test or the Wilcoxon signed-rank test was used, depending on the normality of the data distribution. Pearson’s correlation analysis was performed to evaluate the relationships between clinical outcomes and radiologic measurements. To assess the intra-observer reproducibility and inter-observer reliability of BCA and SCA, an agreement was quantified using the intraclass correlation coefficient (ICC). Two independent researchers (an orthopedic spine surgeon with 1 year of experience and an orthopedic resident with 3 years of experience) performed two series of BCA and SCA measurements on the CT scans.

## 3. Results

Of the 36 patients, 11 were female and 25 were male, with an average age of 64.06 years (range: 41–81 years). No perioperative complications related to the surgery were reported. OPLL was observed in all 36 patients at the C5–6 level, followed by C4–5 (91.7%), C3–4 (69.4%), and C6–7 (58.3%) (Table 1). All patients underwent decompression surgery from C3 to C6, with 15 patients receiving laminoplasty at C3 and 21 patients undergoing C3 laminectomy. The surgeries were performed by two experienced surgeons using the same standardized technique, as described in the surgical technique section.

### 3.1. Radiologic Outcomes

**Figure 2:**
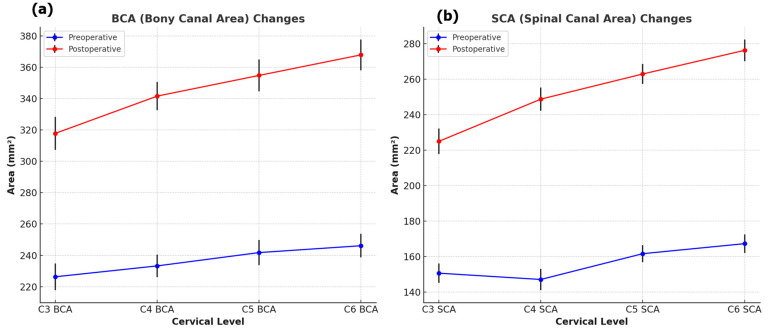
(**a**) Changes in the bony canal area (BCA) preoperatively and postoperatively. (**b**) Changes in the spinal canal area (SCA) preoperatively and postoperatively.

In the analysis of cervical alignment before and after surgery, CL significantly decreased from 15.54 ± 8.99 degrees to 12.49 ± 6.93 degrees (*p* = 0.005). CSVA changed from 22.12 ± 11.11 mm to 23.89 ± 10.80 mm, although this change was not statistically significant (*p* = 0.341). The results of cross-sectional area measurements of the BCA, SCA, and OPLL are presented in Table 2. Both BCA and SCA showed significant increases after surgery at all levels (Figure 2). The average BCA across all levels increased from 239.12 ± 29.48 mm^2^ to 351.19 ± 39.51 mm^2^, representing a change of 112.07 mm^2^ (46.87%). Similarly, the average SCA increased from 157.35 ± 32.13 mm^2^ to 257.85 ± 44.19 mm^2^, with a change of 100.50 mm^2^ (63.87%).

The inter-observer and intra-observer ICC for the measurement of BCA and SCA were above 0.9, ranging from 0.936 to 0.961, indicating excellent intra-observer reproducibility and inter-observer reliability (Table 3).

### 3.2. Clinical Outcomes

In the analysis of clinical outcomes before and two years after surgery, the JOA score improved from 11.97 ± 3.54 to 12.30 ± 3.52, and the NDI improved from 28.63 ± 21.44 to 23.07 ± 20.39, although these improvements were not statistically significant (*p* = 0.587 and *p* = 0.179, respectively). NRS-neck, NRS-R, and NRS-L scores also showed improvements, although these improvements were not statistically significant (Table 4). According to the surgical method, the degree of NRS-neck improvement was compared between the C3 laminoplasty group (*n* = 15) and the C3 laminectomy group (*n* = 21). No significant difference was found between the two groups (0.52 ± 3.50 vs. 1.14 ± 3.25, *p* = 0.804).

**Figure 3:**
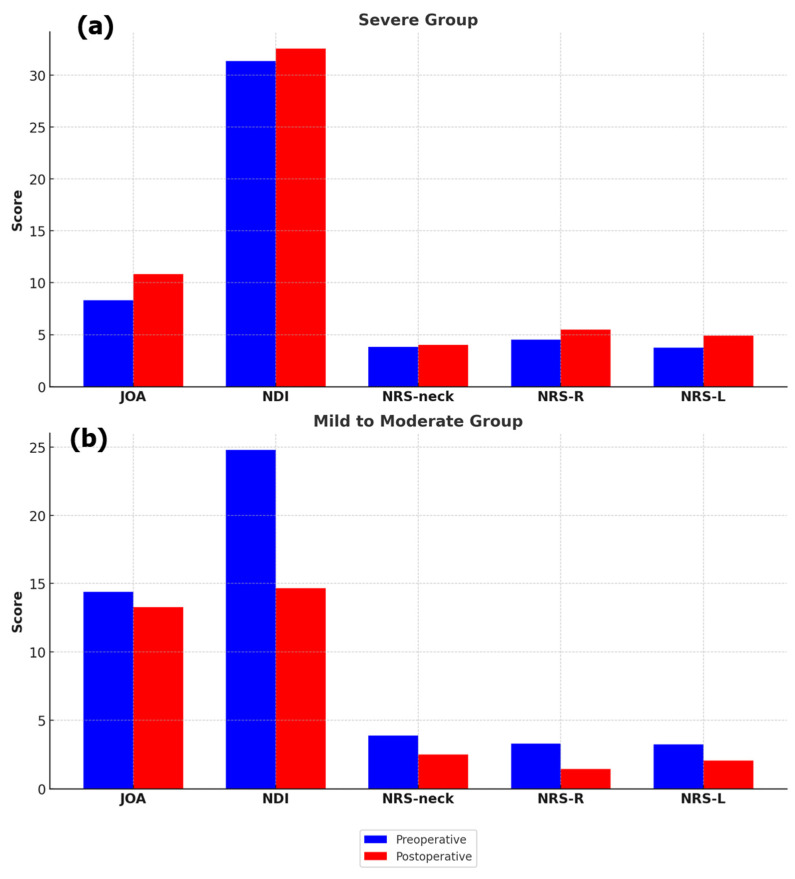
(**a**) Changes in clinical metrics, including JOA score, NDI, NRS-neck, NRS-R, and NRS-L, preoperatively and postoperatively in the severe group. (**b**) Changes in the same clinical metrics preoperatively and postoperatively in the mild to moderate group.

Clinical outcomes were further analyzed by classifying patients according to the severity of myelopathy using the JOA score (JOA score of 11 or less = severe; JOA score of 12 or more = mild to moderate) (Table 5). In the severe group, the JOA score significantly improved from 8.33 ± 2.23 to 10.83 ± 3.69 after surgery (*p* = 0.009). In contrast, in the mild to moderate group, NDI (*p* = 0.010), NRS-neck (*p* = 0.047), and NRS-R (*p* = 0.038) scores showed significant improvements (Figure 3).

### 3.3. Correlations Between Radiologic and Clinical Outcomes

The correlation between radiological canal expansions and clinical outcomes is presented in Table 6. Improvement of NRS-neck showed significant correlations with expansions of both BCA (r = 0.533, *p* = 0.001) and SCA (r = 0.537, *p* = 0.001). Improvement in NDI also showed a significant correlation with BCA expansion (r = 0.351, *p* = 0.045). However, improvement in JOA scores showed no significant correlation with the expansion of BCA or SCA (*p* = 0.265 and 0.292, respectively). Similarly, NRS-R and NRS-L were not associated with canal expansion (all *p*-values > 0.05).

## 4. Discussion

Management strategies for OPLL vary according to symptom severity; asymptomatic or mildly symptomatic patients can be managed conservatively, whereas patients with neurological manifestations typically require surgical decompression through anterior, posterior, or combined approaches [1,20]. Among the various surgical options, open-door laminoplasty has demonstrated efficacy in treating OPLL-associated cervical myelopathy when appropriate patient selection criteria are met [21,22,23]. Recent advances have introduced minimally invasive open-door laminoplasty techniques, which have shown superior outcomes in reducing postoperative axial symptoms compared to conventional expansive open-door laminoplasty [24]. The present study validates these findings while investigating the correlations between clinical symptom recovery (including axial neck pain, radiculopathy, and myelopathy), the extent of spinal canal decompression, and alterations in cervical sagittal alignment.

In the analysis of radiologic outcomes, cervical lordosis was reduced before and after surgery. This reduction in lordosis has also been observed in previous studies [21,25,26]. It is thought to be related to the detachment of cervical extensor muscles attached to the spinous process and resection of the interspinous ligament during surgery [27]. A recent study reported that risk factors for decreased cervical lordosis after laminoplasty differ between cervical spondylotic myelopathy and OPLL. Preoperative CSVA was identified as an independent risk factor in OPLL patients, suggesting that greater preoperative CSVA should be carefully considered in these cases [28].

Studies on the size and compression of the cervical spinal canal have been conducted. Lee et al. [29] measured the spinal canal diameter from C3 to C7 in 469 cadavers and reported a range of 9 to 20.9 mm, with a median diameter of 14.4 mm. The Torg–Pavlov ratio, one of the most common methods, compared the sagittal diameter of the spinal canal with the width of the vertebral body [30]. More recently, direct measurements of bony and spinal canal areas using CT scans have been reported by Dong et al. [10]. They described the volume-occupying rate of the spinal canal by obtaining the area through direct measurement and then used an integration formula to calculate the volume-occupying rate. They concluded that the volume-occupying rate had a significantly higher correlation with JOA scores than the sagittal diameter of the secondary cervical spinal canal and the effective cervical spinal canal ratio.

However, specific studies focused on the relationship between the spinal canal and OPLL are lacking [11,31]. Wang et al. [31] measured the amount of SCA expansion in 82 patients with cervical myelopathy after open-door laminoplasty and found an increase of 123.01 ± 17.06 mm^2^. Park et al. [11] have described a bony spinal canal dimension that increased from 204.3 to 331.7 mm^2^ after a single open-door laminoplasty. Similarly, in our study, the average BCA increased from 239.12 mm^2^ to 351.19 mm^2^ (46.9%), and the average SCA increased from 157.35 mm^2^ to 257.85 mm^2^ (63.9%).

Neurologically, conservative management is generally indicated for patients maintaining JOA scores ≥ 15, with most spine surgeons considering JOA scores of 12–13 as the surgical intervention threshold [32]. While overall cohort analysis revealed no significant differences in pre- and postoperative JOA scores, stratified analysis by myelopathy severity demonstrated significant postoperative improvement in the severe group (8.33 vs. 10.83, *p* < 0.05) but not in the mild-to-moderate group. Similarly, initial analysis of the entire cohort showed no significant differences in NDI, NRS-neck, NRS-R, and NRS-L scores between pre- and postoperative states. However, subgroup analysis revealed significant improvements in NDI (31.33 vs. 32.55, *p* < 0.05) and NRS-neck scores (3.89 vs. 2.50, *p* < 0.05) within the mild-to-moderate group. These findings suggest two important clinical implications. First, clinical outcomes following surgical intervention for OPLL appear to be dependent on preoperative myelopathy severity. Second, contrary to previous reports suggesting axial neck pain as a potential complication of open-door laminoplasty in cervical myelopathy [33,34], our findings demonstrate improvements in both NDI and NRS-neck scores postoperatively.

Although OPLL patients may initially present as asymptomatic, they frequently experience axial neck pain or radicular symptoms prior to developing myelopathic manifestations [35]. During early-stage myelopathy, clinical deterioration may be more readily detected through NRS or NDI scores rather than JOA scores, which are more specific to myelopathy. Meanwhile, neurological recovery has been identified as an independent factor significantly associated with the reduction in postoperative neck pain in patients with cervical myelopathy due to OPLL who underwent cervical spine surgery [36]. Additionally, recent studies suggest that axial neck pain does not necessarily worsen after laminoplasty when appropriate patient selection and surgical techniques are applied [37,38].

These results were consistent with the observed relationship between radiologic and clinical outcomes. The degree of BCA expansion was found to be related to improvements in neck pain, as reflected by NDI and NRS-neck scores. In contrast, myelopathy symptoms, as represented by JOA scores, did not show a significant correlation with canal expansion. Consequently, canal expansion appears to be more closely associated with neck pain than with neurological symptoms.

Previous studies investigating French-door laminoplasty in degenerative cervical myelopathy have reported that insufficient spinal cord expansion following adequate decompression may predict suboptimal neurological recovery [39]. However, evidence explaining the relationship between spinal canal expansion and the improvement of clinical outcomes remains limited. Our findings suggest that alleviation of spinal canal compression may primarily contribute to the improvement of early myelopathic symptoms, particularly neck pain and functional disability. The lack of significant correlation between JOA scores and canal expansion in our study may be attributed to the disproportionate distribution of myelopathy severity in our cohort (mild-moderate: *n* = 22; severe: *n* = 14). Based on these observations, laminoplasty may be indicated not only for spinal canal decompression in severe myelopathy but also for the amelioration of neck pain and functional disability in patients with OPLL-associated myelopathy.

This study has several limitations. First, the sample size was relatively small, with only 36 patients, due to the strict inclusion criteria. In addition, the severity of myelopathy in the included patients varied according to JOA scores. Further studies with larger cohorts are needed to validate these results, as clinical outcomes may vary significantly depending on the severity of myelopathy and the number of patients in this study was limited. Second, not all patients underwent the same surgical technique. C3 laminectomy was performed in 21 patients, while laminoplasty was performed on C3 in 15 patients. Some authors have reported that C3 laminoplasty can affect axial neck pain postoperatively [13,14,37]. However, in our subgroup analysis, there was no significant difference in neck pain between the C3 laminoplasty group and the laminectomy group. Third, long-term follow-up results were not analyzed. This study evaluated clinical scores two years after surgery, while previous studies have reported that recovery in patients with cervical myelopathy typically reaches a plateau between 6 months and 1 year after surgery [40,41].

Despite these limitations, this study is meaningful in that it analyzed the relationships between radiologic measurements and clinical outcomes. In addition to improving myelopathy symptoms in severe cases, cervical open-door laminoplasty could be effective in improving neck pain and NDI through spinal canal expansion.

## 5. Conclusions

Cervical open-door laminoplasty is an effective surgical technique for increasing the bony canal and spinal canal area in patients with OPLL. This study demonstrated that spinal canal expansion improved myelopathy symptoms in severe cases and was significantly correlated with improvements in neck pain and disability. While laminoplasty is typically considered for relieving myelopathy, it may also improve quality of life through the alleviation of neck pain and disability. Further research is needed to validate the long-term clinical benefits and to better understand the relationship between spinal canal expansion and clinical outcomes in OPLL patients.

## Figures and Tables

**Table 1:** Distribution of OPLL in included patients.

Segment	Number (Percentage)
C1–2	3 (8.3%)
C2–3	16 (44.4%)
C3–4	25 (69.4%)
C4–5	33 (91.7%)
C5–6	36 (100.0%)
C6–7	21 (58.3%)
C7-T1	8 (22.2%)

**Table 2:** Radiologic parameters before and after open-door laminoplasty.

		Preoperative	Postoperative	Rate of Change	*p*-Value
CL (°)		15.54 ± 8.99	12.49 ± 6.93	−19.60%	**0.005**
CSVA (mm)		22.12 ± 11.11	23.89 ± 10.80	8.01%	0.341
C3 (mm^2^) *	BCA	226.33 ± 24.40	317.80 ± 25.96	40.41%	**0.001**
	SCA	150.67 ± 39.47	225.00 ± 48.64	49.34%	**0.001**
	OPLL	29.47 ± 27.42	30.47 ± 28.39	3.39%	0.198
C4 (mm^2^)	BCA	233.27 ± 34.72	341.58 ± 38.80	46.43%	**0.000**
	SCA	147.12 ± 36.92	248.79 ± 55.67	69.10%	**0.000**
	OPLL	31.82 ± 25.30	30.48 ± 23.72	−4.19%	**0.014**
C5 (mm^2^)	BCA	241.77 ± 36.51	354.77 ± 49.50	46.74%	**0.000**
	SCA	161.66 ± 38.20	262.97 ± 46.18	62.67%	**0.000**
	OPLL	29.17 ± 17.76	29.17 ± 16.88	0.59%	0.827
C6 (mm^2)^	BCA	246.14 ± 33.90	367.89 ± 54.04	49.46%	**0.000**
	SCA	167.31 ± 42.02	276.31 ± 54.54	65.12%	**0.000**
	OPLL	27.26 ± 17.46	26.60 ± 17.00	−2.41%	0.190
Average (mm^2)^	BCA	239.12 ± 29.48	351.19 ± 39.51	46.87%	**0.000**
	SCA	157.35 ± 32.13	257.85 ± 44.19	63.87%	**0.000**
	OPLL	29.95 ± 15.99	29.53 ± 15.73	−1.41%	0.246

Note: CL—cervical lordosis, CSVA—cervical sagittal vertical axis, BCA—bony canal area, SCA—spinal canal area, OPLL—ossified posterior longitudinal ligament. * 15 patients undergoing C3 laminoplasty except C3 laminectomy. Statistically significant values in bold.

**Table 3:** Inter-class correlation coefficient for bony canal area and spinal canal area measurements.

	Inter-ICC	95%CI	Intra-ICC	95%CI
BCA	0.936	0.909–0.956	0.948	0.912–0.962
SCA	0.961	0.942–0.973	0.956	0.919–0.971

Note: BCA—bony canal area, SCA—spinal canal area, ICC—inter-class correlation coefficient.

**Table 4:** Clinical outcomes before and after open-door laminoplasty.

	Preoperative	Postoperative	*p*-Value
JOA	11.97 ± 3.54	12.30 ± 3.52	0.587
NDI	28.63 ± 21.44	23.07 ± 20.39	0.179
NRS-neck	3.94 ± 2.65	2.97 ± 2.91	0.051
NRS-R	3.97 ± 3.07	3.53 ± 3.58	0.493
NRS-L	3.38 ± 3.16	3.25 ± 3.57	0.852

Note: JOA—Japanese orthopedic association score, NDI—neck disability index, NRS-neck—numeric rating scale of neck pain, NRS-R—numeric rating scale of right arm pain, NRS-L—numeric rating scale of left arm pain.

**Table 5:** Clinical outcomes before and after surgery according to severity of myelopathy.

	Severity *	Preoperative	Postoperative	*p*-Value
JOA	Severe (*n* = 14)	8.33 ± 2.23	10.83 ± 3.69	**0.009**
	mild to moderate (*n* = 22)	14.39 ± 1.61	13.28 ± 3.14	0.161
NDI	Severe (*n* = 14)	31.33 ± 20.50	32.55 ± 23.71	0.582
	mild to moderate (*n* = 22)	24.81 ± 20.32	14.65 ± 12.22	**0.010**
NRS-neck	Severe (*n* = 14)	3.83 ± 2.79	4.00 ± 3.54	0.693
	mild to moderate (*n* = 22)	3.89 ± 2.54	2.50 ± 2.56	**0.047**
NRS-R	Severe (*n* = 14)	4.50 ± 3.12	5.50 ± 3.80	0.248
	mild to moderate (*n* = 22)	3.31 ± 2.44	1.43 ± 1.97	**0.038**
NRS-L	Severe (*n* = 14)	3.75 ± 3.31	4.92 ± 4.36	0.421
	mild to moderate (*n* = 22)	3.25 ± 2.98	2.06 ± 2.62	0.211

Note: JOA—Japanese orthopedic association score, NDI—neck disability index, NRS-neck—numeric rating scale of neck pain, NRS-R—numeric rating scale of right arm pain, NRS-L—numeric rating scale of left arm pain. * A JOA score of 11 or less was classified as severe, and a score of 12 or more was classified as mild to moderate. Statistically significant values in bold.

**Table 6:** Correlations between radiologic changes and clinical outcomes.

	Variables	Pearson’s Correlation Coefficient	*p*-Value
∆JOA	∆BCA	−0.210	0.265
	∆SCA	−0.199	0.292
	Preoperative OPLL	0.038	0.842
	∆CL	0.254	0.176
	∆CSVA	−0.003	0.989
∆NDI	∆BCA	0.351	**0.045**
	∆SCA	0.279	0.116
	Preoperative OPLL	−0.089	0.623
	∆CL	−0.018	0.921
	∆CSVA	0.009	0.962
∆NRS-neck	∆BCA	0.533	**0.001**
	∆SCA	0.537	**0.001**
	Preoperative OPLL	−0.077	0.667
	∆CL	−0.074	0.677
	∆CSVA	−0.089	0.617
∆NRS-R	∆BCA	−0.002	0.990
	∆SCA	−0.070	0.699
	Preoperative OPLL	−0.048	0.789
	∆CL	0.058	0.748
	∆CSVA	0.170	0.344
∆NRS-L	∆BCA	−0.114	0.526
	∆SCA	−0.187	0.297
	Preoperative OPLL	0.055	0.761
	∆CL	0.267	0.133
	∆CSVA	0.186	0.301

Note: JOA—Japanese orthopedic association score, NDI—neck disability index, NRS-neck—numeric rating scale of neck pain, NRS-R—numeric rating scale of right arm pain, NRS-L—numeric rating scale of left arm pain, BCA—bony canal area, SCA—spinal canal area, OPLL—ossification of posterior longitudinal ligament, CL—cervical lordosis, CSVA—cervical sagittal vertical axis. Statistically significant values in bold.

## Data Availability

Original data will be made available upon reasonable request.
